# Biobank – the key to personalized medicine

**DOI:** 10.1590/1516-3180.2022.1405.12072022

**Published:** 2022-09-19

**Affiliations:** I MD. Attending Physician, Thoracic Surgery Service, Lung Transplant Group, Instituto do Coração, Hospital das Clinicas HCFMUSP, Faculdade de Medicina, Universidade de Sao Paulo, Sao Paulo, SP, BR.; II MD, PhD. Full Professor, Thoracic Surgery Program, Instituto do Coração, Hospital das Clinicas HCFMUSP, Faculdade de Medicina, Universidade de Sao Paulo, Sao Paulo, SP, BR; Director, Scientific Department, Associação Paulista de Medicina, São Paulo (SP), Brazil.; Associação Paulista de Medicina, Scientific Department, São Paulo, SP, Brazil

Personalized medicine can be defined as a medical approach based on individualized information that allows planning and treatment considering the patient’s clinical and genetic characteristics. Despite the great advance since the creation of the Human Genome Project aimed at identifying all the genes of human beings, the transcription of the gene was found to be not solely responsible for the result of our organism’s responses. Therefore, the investigations of proteomics and metabolomics projects were also initiated, considering the transcription from deoxyribonucleic acid (DNA) to ribonucleic acid (RNA), its production of proteins and products of cellular metabolism.^1^ Moreover, this data set needs to be associated with the patient’s clinical information. The collection and storage of biological material enable a series of studies that will allow the distinction between the healthy and pathological tissues and the genotype and associated phenotypes.^2^

The biobank is an organized collection of human biological material and associated information, collected and stored for research purposes, according to regulations or pre-defined technical, ethical, and operational standards, under the institutional responsibility and management of the stored materials, with no commercial purposes. In other words, it is the democratic storage of biological materials for research on patients, guaranteed by an institution. Contrarily, the biorepository is the storage of biological material during a specific research project, under institutional responsibility and the researcher’s management.^3^

Motivations for building a biobank are related to new goals of modern medicine and multidisciplinary practice, such as personalized medicine, early diagnosis of specific diseases like cancer or genetic diseases, and response prediction to targeted therapy with the principles of pharmacogenetics.^4,5^

Biobank’s biological materials are valued in terms of the digital traceability of information and presence of clear consent and assent of participants and their guardians (in case of minors), in addition to the technique of sample acquisition, storage, quality control, data collection, correlation to clinical data, data security and access.^6^ Ischemia time clearly changes gene expression. Furthermore, the method of tissue fixation can change the results of laboratory tests, resulting into false positives or negatives. Therefore, in the biobank, best practices must be strictly followed.

Several specimens allow storage, like organs, solid tissues, tumors, umbilical cord, feces, blood and its derivatives, urine, cerebrospinal fluid, teeth, saliva, bronchoalveolar lavage fluid, and perfusates (samples from organ perfusion). Samples must be stored based on the objective to be studied with fresh frozen tissue; since specific preparations are required for extracting DNA, RNA, mitochondria, specific proteins, among others.^2^

However, the biobank is not restricted to the acquisition and storage of biological materials. The progressive use of artificial intelligence and databases, with the possibility of intersecting the collected materials, retrospectively or prospectively, with associated donor information, pushes new frontiers of knowledge. Additionally, the participants can include their own data using the biobank’s websites or Apps, and can be notified about the usage of their information and material.^7^

One of the main biobanks in the world is the United Kingdom (UK) Biobank, managed by England. Currently, it has more than 500,000 participants. Since 2014, magnetic resonance imaging and other images are also being collected. This biobank assesses genetics, physical activities, imaging tests, biomarkers, physical measurements, cognition, and hearing, in addition to prospective questionnaires. Although this large biobank is not compatible with the representation of the general population, as it is a cohort of healthy people, evaluation of public health outcomes can be ideally performed.^8^

Franklin Delano Roosevelt, former United States President, died in 1945 after an acute myocardial infarction. He encouraged his successor Harry Truman to develop the National Heart, Lung, and Blood Institute. In 1948, Truman included the first patient in the institute from one of the main cohorts in the world: the Framingham Heart Study. This cohort allowed several publications and guided the consensus on cardiovascular risk and its prevention. This was possible because of the integration of information and storage of biological materials. This is the concept of a biobank.^9^

In 2021, 89 biobanks in Brazil had been authorized by the National Research Ethics Commission for the most diverse purposes of collecting biological materials. Based on this tool in the arsenal of translational research, worldwide and in Brazil, the Heart Institute of the Medical School of the University of São Paulo (Instituto do Coração da Faculdade de Medicina da Universidade de São Paulo [InCor HCFMUSP]) created the Institutional Biobank, with a capacity for more than 84,000 samples, including the cardiopulmonary department, in which groups can store samples of selected lines of action, such as lung transplantation.^10^

Presently, the concept of evidence-based medicine is developing into medicine based evidence, which means, with the acquisition of new information from large databases, associated with materials collected from biobanks and the integration of artificial intelligence, either by deep learning, neural network, or other forms of algorithms. The evidence, that is, the data, will be able to better predict the treatment, target therapy, specific diagnoses, and others. Evidence-based medicine supports personalized medicine, and the biobank plays a key role in this regard. The collection of biological materials and the reproducibility of the technique in different general and specific populations will allow these studies.

Specific institutions created for biobank such as the UK Biobank or well-structured educational institutions such as the InCor, with the capacity to store information and biological materials, will be able to collect and provide researchers and the population with answers that is lacking at present, especially focused on the patient’s individuality.
